# Population estimates and determinants of severe maternal thinness in India

**DOI:** 10.1002/ijgo.13940

**Published:** 2021-11-01

**Authors:** Tashi Choedon, Vani Sethi, Ranadip Chowdhury, Neena Bhatia, Konsam Dinachandra, Zivai Murira, Arti Bhanot, Dinesh Baswal, Arjan de Wagt, Madhavi Bhargava, Indrapal Ishwarji Meshram, Giridhara R. Babu, Bharati Kulkarni, Hema Divakar, Chandni Maria Jacob, Sarah Louise Killeen, Fionnuala McAuliffe, Ruby Alambusha, William Joe, Mark Hanson

**Affiliations:** ^1^ Institute of Economic Growth New Delhi India; ^2^ Nutrition Section United Nations Children’s Fund New Delhi India; ^3^ Centre for Health Research and Development Society for Applied Studies New Delhi India; ^4^ NITI Aayog Government of India New Delhi India; ^5^ Independent Consultant New Delhi India; ^6^ Regional Office for South Asia UNICEF Kathmandu Nepal; ^7^ Programmes Mamta Health Institute for Mother and Child New Delhi India; ^8^ Department of Community Medicine Yenepoya Medical College Mangalore India; ^9^ Indian Council of Medical Research National Institute of Nutrition Hyderabad India; ^10^ Department of Epidemiology Indian Institute of Public Health Bengaluru India; ^11^ Divakars Specialty Hospital Bengaluru India; ^12^ NIHR Southampton Biomedical Research Centre University Hospital Southampton Southampton UK; ^13^ Institute of Developmental Sciences University of Southampton Southampton UK; ^14^ UCD Perinatal Research Centre School of Medicine University College Dublin National Maternity Hospital Dublin Ireland

**Keywords:** consequences of severe thinness, India, postpartum, pregnancy, prevalence, risk factors for severe thinness, severe thinness

## Abstract

**Objective:**

To examine prevalence, risk factors, and consequences of maternal severe thinness in India.

**Methods:**

This mixed methods study analyzed data from the Indian National Family Health Survey (NFHS)‐4 (2015–2016) to estimate the prevalence of and risk factors for severe thinness, followed by a desk review of literature from India.

**Results:**

Prevalence of severe thinness (defined by World Health Organization as body mass index [BMI] <16 in adult and BMI for age *Z* score < –2 *SD* in adolescents) was higher among pregnant adolescents (4.3%) compared with pregnant adult women (1.9%) and among postpartum adolescent women (6.3%) than postpartum adult women (2.4%) 2–6 months after delivery. Identified research studies showed prevalence of 4%–12% in pregnant women. Only 13/640 districts had at least three cases of severely thin pregnant women; others had lower numbers. Three or more postpartum women aged ≥20 years were severely thin in 32 districts. Among pregnant adolescents, earlier parity increased odds (OR 1.96; 95% CI, 1.18–3.27) of severe thinness. Access to household toilet facility reduced odds (OR 0.72; 95% CI, 0.52–0.99]. Among mothers aged ≥20 years, increasing education level was associated with decreasing odds of severe thinness (secondary: OR 0.74; 95% CI, 0.57–0.96 and Higher: OR 0.54; 95% CI, 0.32–0.91, compared with no education); household wealth and caste were also associated with severe thinness.

**Conclusion:**

This paper reveals the geographic pockets that need priority focus for managing severe thinness among pregnant women and mothers in India to limit the immediate and intergenerational adverse consequences emanating from these deprivations.

## Synopsis

This article reveals the geographic pockets in India that need priority focus for managing severe thinness among pregnant and postpartum women to limit the immediate and intergenerational adverse consequences emanating from these deprivations.

## 1 INTRODUCTION

In most low‐ and middle‐income countries (LMICs), severe thinness—defined by the World Health Organization (WHO) as body mass index (BMI, calculated as weight in kilograms divided by height in meters squared) below 16^1^—among women of reproductive age (15–49 years) continues to persist in selected regions, along with an increasing prevalence of overweight and obesity. Prevalence of severe thinness is estimated at 1.8% among women aged 20–49 years across 60 LMICs.^2^ This estimate is consistent over a 10‐year time frame for most LMICs. In India, the prevalence of thinness or severe thinness ranges between 2% and 41%.^3^, ^4^, ^5^, ^6^, ^7^, ^8^, ^9^, ^10^, ^11^, ^12^

In a study on Asian adult men and women, mortality risk was twice as high among those with very low BMI (<15) compared with very high BMI (>35).^13^ Thinness, either mild (BMI 16–18.49) or severe (BMI <16) in pregnant women increases the risk of preterm birth, small for gestational age (SGA) neonates, low birthweight (LBW), and infant mortality.^14^, ^15^, ^16^, ^17^ However, there is limited evidence on the burden of and risk factors for severe thinness in pregnancy in India. Therefore, it is crucial to understand the prevalence of and risk factors for severe thinness in pregnancy to develop context‐specific preventative policies.

Using data from the National Family Health Survey (NFHS)‐4^18^ and a desk review of literature with a focus on India, the aim of the present study was to examine prevalence, risk factors, and consequences of maternal severe thinness.

## 2 MATERIALS AND METHODS

The geographic scope of the current study is India. The study used a mix of analytical methods including a review of the literature on the prevalence and consequences of severe thinness, and secondary analysis of NFHS‐4^18^ to estimate the prevalence of and risk factors for maternal severe thinness.

### 2.1 Review of literature

We undertook a desk review of papers published in India between January 2010 and December 2019. Papers were searched in the PubMed electronic bibliographic database using the search terms: pregnant*, undernutrition, thin*, severely thin, low BMI, and India, using a time limit of 10 years (2010–2019). A standardized Microsoft Excel version 13 (Microsoft Corp) data extraction template was used to extract data including prevalence, causes, and consequences, with details of date published, authors, type of study, location, duration, and outcomes of interest.

### 2.2 Secondary analyses of NFHS‐4 (2015–2016)

**FIGURE 1:**
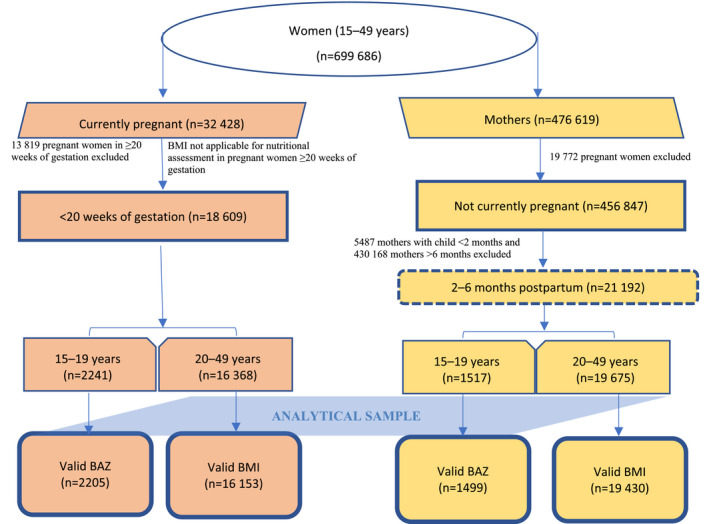
Sampling flow chart for pregnant women (<20 weeks of gestation) and mothers in the postpartum period (2–6 months), National Family Health Survey‐4 (2015–2016)^18^

The prevalence and determinants of severe thinness in pregnancy as well as in the postpartum period (2–6 months) were estimated through analysis of NFHS‐4.^18^ The NFHS survey followed a two‐stage, stratified cluster sample in which there were 699 686 women of reproductive age (15–49 years) selected from a random sample of 576 318 households. The survey yielded an analytical sample of pregnant adolescents and women (*n* = 2205, 15–19 years; *n* = 16 153, 20–49 years, respectively) and postpartum adolescents and women (*n* = 1499, 15–19 years; *n* = 19 430, 20–49 years, respectively) used in the present analysis. Inclusion criteria for pregnant women in the analytical sample for the present analysis comprised the following: (1) <20 weeks of gestation (based on reported gestational age) to avoid misclassification based on BMI cut‐offs; (2) height and weight measurements available for calculating BMI; and (3) BMI not flagged as invalid in the NFHS, i.e. BMI is neither <12 nor >90. Among postpartum women, those under 2 months after delivery were excluded to avoid misclassification based on BMI cut‐offs; a maximum of 6 months after delivery was adopted as the cut‐off to avoid recall bias about the most recent pregnancy with past pregnancies (Figure 1).

### 2.3 Variables

Adult BMI cut‐offs for thinness (<18.5) and severe thinness (<16) as per WHO were used to estimate the prevalence of thinness among pregnant and postpartum women (20–49 years).^19^ For adolescent females (15–19 years) in the sample, BMI for age *Z* score (BAZ) < –1 standard deviation (*SD*) and <–2 *SD* were used to estimate the prevalence of thinness and severe thinness, as BAZ < –2 *SD* corresponded to BMI of 16.5 as per a recently published analysis of married adolescents under NFHS‐4.^20^ Disaggregation of severe thinness prevalence estimates either by age or state was not possible due to the small sample size. However, we present the distribution of several cases by age and district.

Based on existing literature, independent variables used in the analysis consisted of personal and household characteristics. Personal characteristics included maternal age (defined in 5‐year age groups as 20–24, 25–29, 30–34, and ≥35 years); age at marriage <18 years or not; parity as 0, 1, 2, 3 or more; and education as no, primary, secondary, and higher. Household characteristic included caste, access to drinking water, access to toilets, and wealth quintiles.^20^ Additionally, for pregnant women or adolescents, variables related to diet such as consumption of milk/curd, pulses/beans, eggs/meat/fish, and dark green leafy vegetables daily, and weekly consumption of fried food and aerated drinks were added. For postpartum women, variables related to diet and access to antenatal care services were added.

Severe thinness and severe underweight are used interchangeably and for this article they mean the same.

### 2.4 Statistics analysis

National level sampling weight was used during the analysis to maximize the representativeness of the study population. Descriptive analyses were conducted to present characteristics of the study sample for women in pregnancy and the postpartum period. We developed maps depicting district‐wise cases to study the variability in the prevalence of severe thinness at the state level. Two logistic regression analyses were carried out to examine the associations between severe thinness and its correlates after adjusting for other covariates, including gestational age.

Data were analyzed using Stata version 15.1 (StataCorp LLC, College Station, TX, USA). *P* < 0.01, *P* < 0.05, and *P* < 0.10 were considered statistically significant.

## 3 RESULTS

Table 1 presents the research studies on the prevalence of thinness and/or severe thinness among pregnant women in community and facility settings in India and its consequences in the last decade (2010–2019). The study sites were from nine states of India. Only one of the 10 studies identified, conducted in Andhra Pradesh and Telangana in 2017, provided exclusive estimates for severe thinness based on mid‐upper arm circumference (MUAC) <21 cm and the prevalence ranged from 4% to 12%.^6^ The other studies provided estimates of thinness including severe thinness, but the definitions for measuring thinness were inconsistent. Thinness, including severe thinness, was measured using BMI <18.5 in five studies,^3^, ^4^, ^5^, ^7^, ^10^ BMI <19.9 in three studies,^8^, ^9^, ^11^ and BMI 19.8 as thinness and BMI <18.5 as severe thinness in one study.^12^ Only one study measured severe thinness using MUAC <21 cm and thinness using MUAC <23 cm.^6^

**TABLE 1:** Research on prevalence, determinants, and consequences of maternal thinness and severe thinness in the last decade (2010–2019) in India

Study	Author	Year published	Location	Design	Sampling and sample size	Duration	Outcomes
1.	Mukherjee et al.^3^	2019	ICDS centers, 3 districts of West Bengal	Community‐based intervention study	182 pregnant women (first trimester)	10 months (April 2018–February 2019)	Prevalence (from both groups): 30.3% severely thin (BMI <16) or thin (18.49), 49% anemic
							Determinants: Not investigated
							Consequences: Prevalence of LBW was highest among severely thin and overweight/obese mothers, at 22% and 20% respectively, followed by thin and normal BMI at 15% and 18%, respectively
2.	Patel et al.^4^	2018	Four districts of eastern Maharashtra (Bhandara, Chandrapur, Nagpur, Wardha). Site for NIH's global network for maternal and newborn registry	Prospective observational cohort study	72 750 women (first trimester pregnant or within 42 days postpartum), actively identified in neighboring areas of primary health centers in 20 study clusters	7.5 years (June 2009–December 2016)	Prevalence: 35.5% thin or severely thin (BMI <18.5), 91% anemic, 32.8% both underweight and anemic
							Determinants: Not investigated
							Consequences: Maternal deaths: 29 (19 only anemia, 8 both anemia and thin, 1 underweight, and 2 obese). Stillbirths 24 vs 21 per 1000 births, neonatal deaths 21.9 vs 18.3, and LBW 19.9 vs 15.6 (underweight vs normal). LBW significantly higher among thin women (RR 1.21 (1.16–1.25)). Risks for still births 1.47 (RR 1.17–1.85), neonatal deaths (RR 1.74 (1.33–2.26)), and LBW (RR 1.49 (1.37–1.62)) highest among those both anemic and thin
3.	Short et al.^5^	2018	Nagpur and Belagavi, India; Thatta, Pakistan (sites for Maternal Newborn Health Registry, Global network. Also includes sites in Africa and Guatemala)	Prospective observational study	41 778 of which 30 342 were from India (pregnant women, study clusters constituting areas near government health centers undertaking 300–500 deliveries annually)	2009 onward (analysis restricted to entries from January 2013 to June 2016, weight recorded at <12 weeks of gestation)	Prevalence: 41% severely thin or thin (<18.5) (India sample)
							Determinants: Individual variables (age, parity) varied by BMI status, but no difference in education, number of antenatal care checkups, and place of delivery
							Consequences: Maternal complications were least prevalent among thin mothers. Adverse maternal outcome: 27.2% underweight mothers vs 47.2% overweight (BMI 25–29) and 56.0% obese (BMI ≥30)
4.	UNICEF, India^6^	2017	Two states: Andhra Pradesh and Telangana Andhra Pradesh: four districts (Kadapa, Krishna, West Godavari, and Vishakhapatnam) across five ICDS projects (three rural and two tribal) Telangana: three districts of Karimnagar, Khammam, and Warangal covering five ICDS projects (three rural and two tribal)	Community‐based cross‐sectional	360 pregnant and lactating women (of infants aged 0–6 months) per state from 60 AWCs 57% pregnant women and 43% lactating MIS records: 520 beneficiaries from 60 AWCs per state per year	Primary data collection: July 2016 and November 2016 Review of MIS (2014–2016) and April–August 2017	Prevalence: Thinness (MUAC <23 cm) 15%–22%; severe thinness (MUAC <21 cm) 4%–12%
							Determinants: Not investigated
							Consequences: Not investigated
5.	Bhavadharini et al.^7^	2017	Three antenatal clinics and private maternity centers in Chennai	Secondary analysis of facility records	2728 pregnant women (in the first trimester)	3 years (January 2011–January 2014)	Prevalence: 5.6% (*n* = 154) severely thin or thin (BMI <18.5), 29% (*n* = 791) normal weight, 18.5% (*n* = 504) overweight, and the rest 46.9% (*n* = 1279) were obese
							Determinants: Not investigated
							Consequences: Thin women gaining less weight than recommended were at higher odds of having LBW babies compared with normal and overweight gaining less weight (underweight OR 2.4; 95% CI, 0.72–8.1, *P* = 0.15; normal weight OR 1.5; 95% CI, 0.8–2.7, *P* = 0.2; overweight OR 0.7; 95% CI, 0.3–1.3, *P* = 0.2) Underweight women gaining more than recommended weight were at higher odds for having LBW babies compared with normal, overweight, and obese women (underweight OR 2.7; 95% CI, 0.2–3.21, *P* = 0.42; normal weight OR 1.4; 95% CI, 0.4–3.1, *P* = 0.5; overweight OR 0.6; 95% CI, 0.2–1.8, *P* = 0.3; obese OR 0.9; 95% CI, 0.6–1.5, *P* = 0.8)
6.	Tellapragada et al.^9^	2016	Manipal (Kasturba Medical College)	Prospective observational cohort study	790 (pregnant women, ≤24 weeks of gestation)	3 years (May 2011–April 2014)	Prevalence: 25% severely thin or thin (BMI cut‐off ≤19.9)
							Determinants: Not investigated
							Consequences: Not investigated
7.	Sebastian et al.^10^	2015	Labor room register, The Christian Medical College and Hospital, Vellore, Tamil Nadu	Prospective observational study	36 674 babies and their mothers, rotational sampling (four quarters of 1 year from 1990 to 2010)	15 years (1990–2010) (weight at delivery taken for calculating BMI)	Prevalence: Thinness (BMI <18.5) 2.51% and 1.41% prior to and after 2004
							Determinants: Not investigated for maternal underweight
							Consequences: Thin women were 1.71 (1.38–2.13) times at higher odds for having SGA babies compared with women who were of normal weight (*P* < 0.001) Anemic mothers had 1.29 (1.01–1.6) times higher odds. Incidence of SGA was 11.4% in 1996 and 8.4% in 2010
8.	Agarwal et al.^8^	2016	Antenatal clinics, Pt. J.N.M Medical College, Raipur, Chhattisgarh	Prospective, observational study	1000 pregnant women (at/before 16 weeks of gestation)	3 years (2011–2014)	Prevalence: 18% (514) severely thin or thin (≤19.9)
							Determinants: Not investigated
							Consequences: Anemia more common in thin women compared with obese (14.9% vs 9.5%, *P* = 0.008)
9.	Jain et al.^11^	2012	Department of Obstetrics and Gynecology, SMS Medical College, Jaipur	Observational study	300 pregnant women (nulliparous women with singleton term pregnancies who were in early labor)	1 year and 3 months (June 2009–September 2010)	Prevalence: 10 (3.3%) were severely thin or thin (BMI ≤19.9), 193 (61%) had normal BMI, 71 (24%) were overweight, and 17 (6%) were obese
							Determinants: Not investigated
							Consequences: LBW more prevalent among thin women (80%) compared with women with BMI 20–25 (21.21%) (OR 0.32; 0.180–0.568)
10.	Kumar et al.^12^	2010	Antenatal clinics, Maulana Azad Medical College, New Delhi	Prospective observational study	2027 pregnant women (before 8 weeks of gestation)	1 year (July 2005–July 2006)	Prevalence: 7.2% had very low BMI (<18.5), 9.7% had low BMI (18.5–19.8), 25.8% high BMI (>26), underweight (weight <40 kg) 2.6%
							Determinants: Not investigated
							Consequences: LBW babies delivered: 35.4% with very low BMI and 33.7% with low BMI compared with 24% in normal BMI group (*r* = 28.1, *P* = 0.001). The risk of having LBW babies in the low BMI group was found to be 1.9 times that of normal BMI (OR 1.950; 95% CI, 1.108–3.431). 37.7% women with weight <40 kg delivered LBW babies compared with normal weight women 26.5% (*n* = 402/1514) (*r* = 25.4, *P* < 0.05)

Abbreviations: AWC, Anganwadi Centre; BMI, body mass index (calculated as weight in kilograms divided by height in meters squared); ICDS, Integrated Child Development Service; LBW, low birthweight; MIS, Management Information System; MUAC, mid‐upper arm circumference; NIH, National Institute of Health; SGA, small for gestational age.

Among the consequences for mothers, maternal death, rate and types of obstetric complications, gestational weight gain, and anemia were investigated.^4^, ^5^, ^7^, ^8^ For child outcomes, LBW was the most investigated outcome.^3^, ^4^, ^5^, ^11^, ^12^ The odds of LBW were 1.7–2 times higher among severely thin or thin mothers compared with mothers of normal BMI.^4^, ^5^, ^11^, ^12^ The majority of the women in the studies were older than 20 years.

### 3.1 Findings from NFHS‐4 analysis

#### 3.1.1 Study sample characteristics

The majority of women were rural residents (*n* = 30 090) and Hindu (*n* = 28 486). Half belonged to the 20–24 years age group. Around one‐third of the population belonged to socially disadvantaged groups (“scheduled caste,” “scheduled tribe,” and “other backward classes,” as defined by the Government of India in NFHS‐4^18^). About 80% had access to improved drinking water which was piped in, but less than half had access to improved toilet facilities. Table 2 summarizes the characteristics of the study sample.

**TABLE 2:** Sample characteristics of pregnant women at <20 weeks of gestation and postpartum women (2–6 months)

Characteristics	Pregnant women		Postpartum mothers	
	15–19 years	20–49 years	15–19 years	20–49 years
	(*n* = 2205) No. (%)	(*n* = 16 153) No. (%)	(*n* = 1499) No. (%)	(*n* = 19 430) No. (%)
Personal characteristics				
Age group, year				
20–24	NA	7847 (50.6)	NA	8774 (47.8)
25–29	NA	5486 (34.3)	NA	6829 (35.1)
30–34	NA	2037 (11.3)	NA	2651 (12.3)
>34	NA	783 (3.8)	NA	1176 (4.9)
Marriage at <18 years				
No	1860 (84.5)	11 548 (70.7)	176 (11.1)	12 825 (63.8)
Yes	345 (15.5)	4605 (29.3)	1323 (88.9)	6605 (36.2)
Gestational age, week				
4	200 (8.9)	1134 (6.3)	NA	NA
8	518 (22.6)	3199 (19.4)	NA	NA
12	512 (23.3)	3964 (25.5)	NA	NA
16	505 (23.3)	3918 (24.2)	NA	NA
20	470 (21.9)	3938 (24.6)	NA	NA
Parity				
0	1847 (84.2)	5415 (34.6)		
1	321 (14.2)	5402 (34.0)	1270 (85.6)	6464 (33.8)
2	36 (1.5)	2869 (17.3)	214 (13.5)	6532 (35.3)
≥3	1 (0.0)	2467 (14.2)	15 (0.9)	6434 (30.9)
Highest educational level				
No education	419 (18.0)	4118 (25.3)	282 (18.3)	5332 (26.6)
Primary	304 (12.9)	2105 (12.1)	203 (11.6)	2709 (13.3)
Secondary	1436 (66.7)	7671 (46.4)	991 (68.1)	9111 (46.4)
Higher	46 (2.4)	2259 (16.2)	23 (1.9)	2278 (13.6)
Occupation: Work in last 12 months				
Unemployed/don't know	308 (87.2)	2076 (81.3)	209 (87.7)	2849 (86.7)
Professional/technical/managerial/clerical	2 (0.4)	72 (3.6)	NA	73 (1.7)
Manual skilled and unskilled	7 (0.9)	107 (3.6)	3 (2.8)	91 (2.0)
Others	53 (11.5)	372 (11.5)	32 (9.5)	413 (9.6)
Has money that respondent alone can decide how to use				
No	256 (68.1)	1635 (60.9)	179 (75.0)	2193 (64.3)
Yes	114 (31.9)	992 (39.1)	65 (25.0)	1233 (35.7)
Has bank or savings account that respondent uses				
No	287 (77.3)	1489 (56.5)	167 (63.9)	1770 (53.1)
Yes	83 (22.7)	1138 (43.5)	77 (36.1)	1656 (46.9)
Knows program in this area that give loans to women to start or expand a business				
No	262 (70.5)	1729 (63.6)	165 (62.0)	2288 (64.7)
Yes	108 (29.5)	898 (36.4)	79 (38.0)	1138 (35.3)
Husband/partner's characteristics				
Education level				
No education	55 (13.0)	407 (15.1)	44 (14.7)	565 (15.6)
Primary	55 (15.0)	352 (12.3)	37 (14.3)	469 (13.1)
Secondary	229 (63.6)	1426 (53.9)	149 (63.7)	1852 (53.4)
Higher	31 (8.4)	442 (18.7)	14 (7.3)	540 (17.9)
Occupation				
Unemployed/don't know	43 (12.2)	149 (6.0)	30 (7.9)	192 (5.6)
Professional/technical/managerial/ clerical	24 (5.5)	310 (13.1)	7 (5.3)	362 (11.1)
Manual‐skilled and unskilled	125 (33.0)	818 (30.6)	72 (35.5)	1123 (34.5)
Others	178 (49.3)	1350 (50.3)	135 (51.3)	1749 (48.8)
Household characteristics				
Place of residence				
Rural	1839 (81.1)	12 067 (70.0)	1235 (78.8)	14 949 (73.0)
Urban	366 (18.9)	4086 (30.0)	264 (21.2)	4481 (27.1)
Household size				
<5	688 (32.4)	6087 (37.7)	285 (19.3)	3964 (20.5)
≥5	1517 (67.6)	10 066 (62.3)	1214 (80.7)	15 466 (79.5)
Religion of the household				
Hindu	1697 (78.8)	11 550 (76.8)	1104 (78.7)	14 135 (79.0)
Muslim	354 (18.2)	2571 (17.5)	240 (16.3)	2956 (16.3)
Other	154 (3.0)	2032 (5.7)	155 (5.0)	2339 (4.7)
Caste or tribe of the household				
Scheduled caste	477 (23.5)	3095 (21.4)	336 (25.7)	3644 (21.2)
Scheduled tribe	383 (10.5)	3040 (9.2)	321 (12.6)	4064 (11.0)
Other backward class	947 (45.0)	6412 (45.4)	548 (38.1)	7534 (44.3)
Other	398 (20.9)	3606 (23.9)	294 (23.5)	4188 (23.4)
Household has a below poverty line card				
No	1110 (48.4)	10 094 (62.3)	768 (52.2)	11 782 (59.8)
Yes	1095 (51.6)	6059 (37.7)	731 (47.8)	7648 (40.2)
Improved source of drinking water				
No	467 (18.5)	2809 (16.5)	400 (27.4)	3835 (19.1)
Yes	1738 (81.5)	13 344 (83.5)	1099 (72.6)	15 595 (80.9)
Improved toilet facilities				
No	1531 (68.5)	9340 (59.6)	1041 (69.4)	11 924 (63.0)
Yes	674 (31.5)	6813 (40.4)	458 (30.6)	7506 (37.0)
Wealth index				
Poorest	674 (29.6)	3693 (22.4)	442 (26.1)	4916 (24.5)
Poorer	637 (27.8)	3562 (20.2)	468 (30.9)	4560 (21.7)
Middle	444 (20.1)	3233 (20.0)	315 (23.8)	3838 (19.3)
Richer	316 (16.3)	2871 (18.2)	192 (14.0)	3253 (18.6)
Richest	134 (6.2)	2794 (19.2)	82 (5.2)	2863 (15.9)
Access to services				
Pregnancy registered				
No	NA	NA	183 (10.4)	2868 (14.0)
Yes	NA	NA	1316 (89.6)	16 562 (86.0)
Antenatal care in first trimester				
No	NA	NA	615 (38.1)	8140 (41.6)
Yes	NA	NA	884 (61.9)	11 290 (58.4)
At least four antenatal care visits				
No	NA	NA	824 (45.8)	10 364 (50.1)
Yes	NA	NA	675 (54.2)	9066 (49.9)
During pregnancy, given or bought iron tablets/syrup				
No	NA	NA	312 (18.1)	4364 (21.4)
Yes	NA	NA	1187 (81.9)	15 066 (78.6)
Consumed at least iron–folic acid 100				
No	NA	NA	1142 (72.0)	13 892 (68.8)
Yes	NA	NA	357 (28.0)	5538 (31.2)
Taken drugs for intestinal worms				
No	NA	NA	1290 (84.8)	16 490 (82.7)
Yes	NA	NA	209 (15.2)	2940 (17.3)
Received supplementary nutrition from Anganwadi center during pregnancy				
No	NA	NA	587 (37.1)	8274 (43.3)
Yes	NA	NA	912 (62.9)	11 156 (56.7)
Benefits received during pregnancy: health and nutrition education				
No	NA	NA	914 (55.2)	12 254 (61.9)
Yes	NA	NA	585 (44.8)	7176 (38.1)
Diet				
Drink milk or curd daily				
No	1363 (57.9)	9142 (52.0)	972 (62.2)	11 622 (55.1)
Yes	842 (42.1)	7011 (48.0)	527 (37.8)	7808 (44.9)
Eat pulses or beans daily				
No	1293 (57.5)	9353 (54.0)	843 (55.6)	11 113 (54.0)
Yes	912 (42.5)	6800 (46.0)	656 (44.4)	8317 (46.0)
Eat eggs/fish/meat daily				
No	2076 (90.0)	15 135 (92.1)	1383 (88.6)	18 131 (92.1)
Yes	129 (10.0)	1018 (7.9)	116 (11.4)	1299 (7.9)
Eat dark green leafy vegetable daily				
No	1174 (52.8)	8394 (53.0)	750 (49.1)	9948 (51.9)
Yes	1031 (47.2)	7759 (47.0)	749 (50.9)	9482 (48.1)
Consume daily milk/curd and pulses/beans or eggs/fish/meat and dark green leafy vegetables				
No	1919 (85.4)	13 637 (81.9)	1286 (82.9)	16 377 (82.0)
Yes	286 (14.6)	2516 (18.1)	213 (17.1)	3053 (18.0)
Eat fried food weekly				
No	1482 (64.0)	10 616 (64.1)	1031 (64.5)	13 101 (64.8)
Yes	723 (36.0)	5537 (35.9)	468 (35.5)	6329 (35.2)
Aerated drinks weekly				
No	1869 (83.6)	13 106 (80.5)	1311 (87.3)	16 366 (83.6)
Yes	336 (16.4)	3047 (19.5)	188 (12.7)	3064 (16.4)
Iodine present in salt				
No	161 (7.8)	946 (6.7)	93 (6.3)	1171 (7.2)
Yes	2044 (92.2)	15 207 (93.3)	1406 (93.7)	18 259 (92.8)
Alcohol/tobacco use				
Drink alcohol				
No	2172 (99.3)	15 844 (99.3)	1484 (99.4)	19 076 (99.2)
Yes	33 (0.7)	309 (0.7)	15 (0.6)	354 (0.8)
Use tobacco				
No	139 (3.4)	1308 (4.4)	1415 (96.9)	17 729 (94.8)
Yes	2066 (96.6)	14 845 (95.6)	84 (3.1)	1701 (5.2)

#### 3.1.2 Prevalence of severe thinness

The national prevalence of thinness/severe thinness was 4.3% (95% CI, 3.4–5.5) in adolescents (15–19 years) and 1.9% (95% CI, 1.6–2.2) in adult pregnant women (≥20 years). A similar pattern in prevalence of severe thinness was observed among postpartum adolescent women (6.3%; 95% CI, 5.0–8.0) and postpartum adult women (2.4%; 95% CI, 2.2–2.8).

**FIGURE 2:**
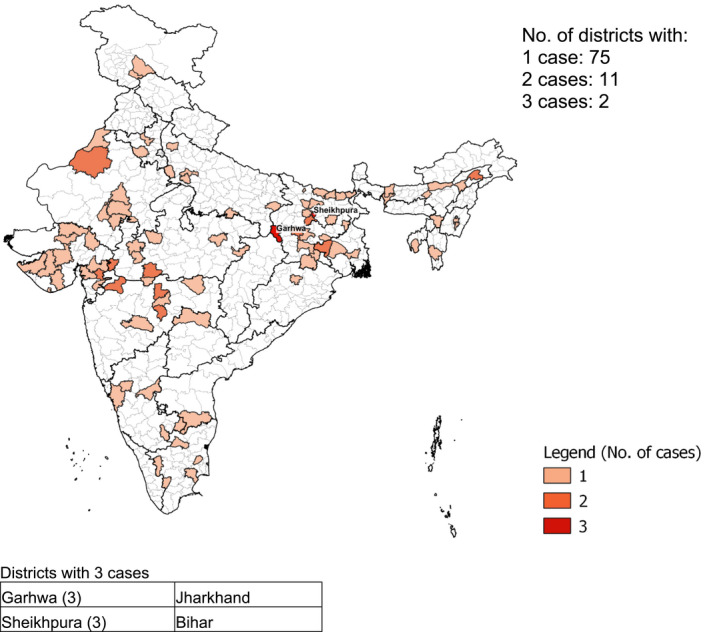
Distribution by district of 103 severely thin (body mass index for age *Z* score < –2 *SD*) pregnant adolescents, India, National Family Health Survey‐4

**FIGURE 3:**
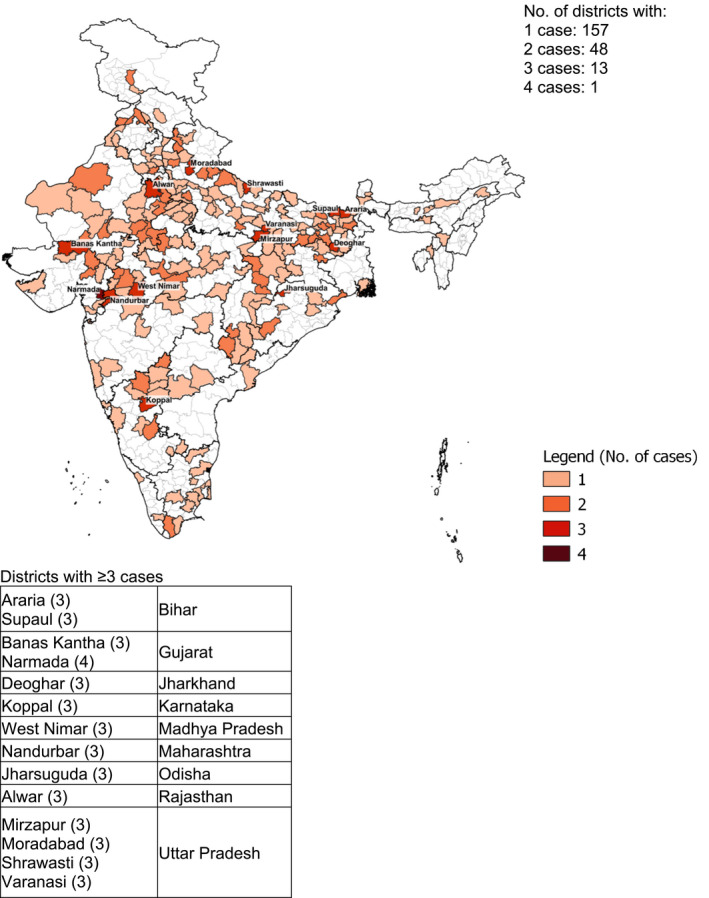
Distribution by district of 296 severely thin (body mass index <16) pregnant women, India, National Family Health Survey‐4

Among pregnant adolescents, 88 out of 640 districts in India had one or more cases of severely thin individuals, with the highest number in Garhwa (Jharkhand) and Shekhpura (Bihar)—three cases each (Figure 2). Cases of severely thin pregnant women aged ≥20 years were spread across 219 out of 640 districts (Figure 3), with the highest number of cases (*n* = 4) in Narmada (Gujarat). There were 13 out of 640 districts with three cases each.

**FIGURE 4:**
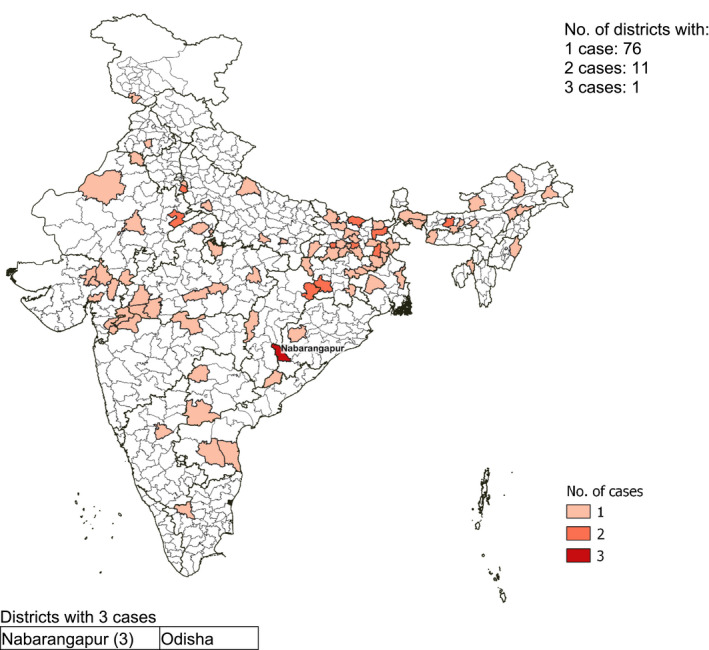
Distribution by district of 101 severely thin (body mass index for age *Z* score < –2 *SD*) adolescent mothers, India, National Family Health Survey‐4. Body mass index for age *Z* score –2 *SD* instead of –3 *SD* was used as the cut‐off for severe thinness among pregnant adolescents, as described in the methods

**FIGURE 5:**
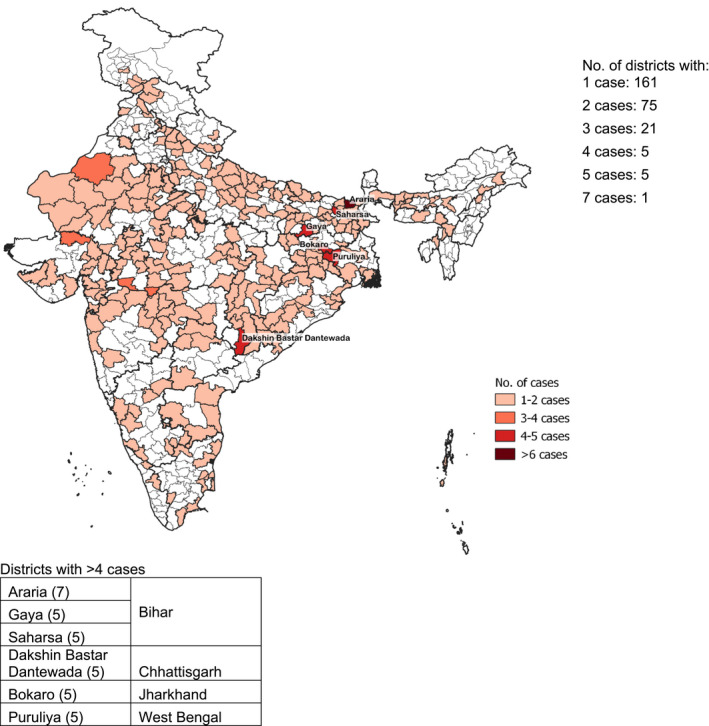
Distribution by district of 2486 severely thin (body mass index <16) mothers aged ≥20 years, India, National Family Health Survey‐4

Among postpartum adolescents, the 101 cases of severe thinness were spread across 88 districts with three cases in Nabarangapur (Odisha) (Figure 4). Among postpartum adult women aged ≥20 years, severe thinness was spread across 268 districts, with the highest number of cases (*n* = 7) in Araria (Bihar) (Figure 5).

#### 3.1.3 Risk factors associated with severe thinness

For a subset analysis among adolescents, logistic regression analysis showed that after adjusting for other factors among pregnant adolescents, as compared with nulliparous pregnant women, having one earlier pregnancy increased the odds of severe thinness by almost 2 times (OR 1.96; 95% CI, 1.18–3.27; *P* < 0.05). However, any other personal or household background characteristics were not observed to be associated with severe thinness among pregnant adolescents. Pregnant adolescents consuming fried foods weekly had lower odds of severe thinness (OR 0.52; 95% CI, 0.32–0.84; *P* < 0.01).

Among pregnant women aged ≥20 years, those aged 25–29 years had lower odds of thinness than the youngest age group of 20–24 years (OR 0.71; 95% CI, 0.52–0.96, *P* < 0.05). The odds of severe thinness were low among women belonging to “scheduled tribe” (OR 0.65; 95% CI, 0.44–0.95, *P* < 0.05) and “general caste” households (OR 0.67; 95% CI, 0.45–0.98, *P* < 0.05) compared with women belonging to “scheduled caste” households.^18^ Access to household toilets was also associated with lower odds of severe thinness (OR 0.72; 95% CI, 0.52–0.99, *P* < 0.05) (Table 3).

**TABLE 3:** Adjusted odds ratios of severe thinness among pregnant adolescents/women at <20 weeks of gestation, India (National Family Health Survey‐4)

	Adolescents (15–19 years) (*n* = 1634)	Adult women (20–49 years) (*n* = 9418)
	BAZ < –2 *SD*, OR (95% CI)	BMI <16, OR (95% CI)
Personal background		
Age group, year		
20–24^a^		
25–29		0.71 (0.52–0.96)^c^
30–34		0.73 (0.46–1.15)
>34		1.09 (0.60–1.98)
Age at marriage under 18 years		
No^a^		
Yes		0.89 (0.66–1.21)
Parity		
0^a^		
1	1.96 (1.18–3.27)^c^	1.38 (1.00–1.90)^c^
2	0.65 (0.08–4.91)	0.99 (0.65–1.54)
≥3	NA	1.37 (0.84–2.26)
Highest educational level		
No education^a^		
Primary	1.11 (0.55–2.24)	0.85 (0.57–1.26)
Secondary	0.95 (0.55–1.66)	0.99 (0.73–1.37)
Higher	0.56 (0.07–4.55)	0.85 (0.50–1.43)
Household background		
Place of residence		
Rural^a^		
Urban	1.54 (0.90–2.65)	1.30 (0.95–1.80)
Caste or tribe of the household		
Scheduled caste^a^		
Scheduled tribe	0.53 (0.25–1.12)	0.65 (0.44–0.95)^c^
Other backward classes	0.99 (0.58–1.69)	0.91 (0.68–1.23)
Other	1.32 (0.71–2.45)	0.67 (0.45–0.98)
Improved source of drinking water		
No^a^		
Yes	1.06 (0.63–1.78)	1.22 (0.88–1.69)
Improved toilet facilities		
No^a^		
Yes	1.15 (0.67–1.98)	0.72 (0.52–0.99)^c^
Wealth index		
Poorest^a^		
Poorer	1.03 (0.59–1.80)	0.82 (0.59–1.13)
Middle	0.82 (0.42–1.61)	0.68 (0.46–0.99)^c^
Richer	0.94 (0.44–2.04)	0.62 (0.39–1.00)^b^
Richest	0.64 (0.22–1.92)	0.62 (0.34–1.10)
Dietary intake		
Consumes daily milk/curd and pulses/beans or eggs/fish/meat and dark green leafy vegetables		
No^a^		
Yes	1.02 (0.56–1.86)	1.21 (0.87–1.69)
Eats fried food weekly		
No^a^		
Yes	0.52 (0.32–0.84)^d^	0.88 (0.68–1.14)
Takes aerated drinks weekly		
No^a^		
Yes	1.65 (0.96–2.85)	1.10 (0.79–1.51)

Abbreviation: BAZ, body mass index for age *Z* score.

^a^ Reference category.

^b^ *P* < 0.10.

^c^ *P *< 0.05.

^d^ *P* < 0.01.

Among postpartum women, the odds of severe thinness were low among women aged 30–34 years (OR 0.45; 95% CI, 0.30–0.70, *P* < 0.01) compared with the youngest group aged 20–24 years. With every level of completed education after primary schooling, odds of severe thinness declined compared with no education (secondary OR 0.74; 95% CI, 0.57–0.96, *P* < 0.05; and higher OR 0.54; 95% CI, 0.32–0.91, *P *< 0.05). As for postpartum women, belonging to “tribal households” (OR 0.62; 95% CI, 0.46–0.83, *P* < 0.01) and “other backward households” (OR 0.72; 95% CI, 0.56–0.93, *P* < 0.05) reduced the odds of severe thinness compared with those belonging to “scheduled caste” households.^18^ Compared with the poorest, mothers belonging to any of the higher wealth index quintiles had lower odds of severe thinness, with odds decreasing consistently with an increase in wealth index‐based quintile. Counterintuitively, among postpartum adolescents, those who reported receiving supplementary food and nutrition education at the *Anganwadi* center had higher odds of severe thinness (OR 1.79; 95% CI, 1.08–2.98, *P* < 0.05) than those who did not, which may be explained by selection bias in service delivery and also those availing of *Anganwadi* services belonging to lower socioeconomic groups (Table 4). All pregnant women and lactating mothers registered with *Anganwadi* centers are eligible for the foods irrespective of nutritional status.

**TABLE 4:** Adjusted odds ratios of severe thinness among adolescents and women aged 20 years or older in the postpartum period, India (National Family Health Survey‐4)

	Adolescents (15–19 years) (*n* = 977)	Adult women (20–49 years) (*n* = 11 153)
	BAZ < –2 *SD*, OR (95% CI)	BMI <16, OR (95% CI)
Personal background		
Age group, year		
20–24^a^		
25–29		0.86 (0.68–1.10)
30–34		0.45 (0.30–0.70)^c^
>34		0.76 (0.47–1.25)
Age at marriage under 18 years		
No^a^		
Yes		1.08 (0.86–1.35)
Parity		
1^a^		
2	0.83 (0.43–1.60)	0.78 (0.60–1.01)
≥3	NA	0.79 (0.58–1.07)
Highest educational level		
No education^a^		
Primary	1.13 (0.58–2.20)	1.01 (0.76–1.34)
Secondary	0.79 (0.46–1.38)	0.74 (0.57–0.96)
Higher	NA	0.54 (0.32–0.91)^b^
Household background		
Place of residence		
Rural^a^		
Urban	1.06 (0.55–2.03)	0.98 (0.72–1.32)
Caste or tribe of the household		
Scheduled caste^a^		
Scheduled tribe	0.97 (0.50–1.88)	0.62 (0.46–0.83)^c^
Other backward classes	1.32 (0.74–2.37)	0.72 (0.56–0.93)^b^
Other	1.04 (0.51–2.13)	0.86 (0.63–1.17)
Improved source of drinking water		
No^a^		
Yes	1.19 (0.72–1.97)	1.03 (0.8–1.32)
Improved toilet facilities		
No^a^		
Yes	0.73 (0.39–1.36)	0.93 (0.71–1.22)
Wealth index		
Poorest^a^		
Poorer	0.68 (0.40–1.16)	0.75 (0.58–0.96)^b^
Middle	0.47 (0.22–0.98)^b^	0.53 (0.38–0.75)^c^
Richer	0.41 (0.16–1.07)	0.55 (0.36–0.83)^c^
Richest	0.98 (0.30–3.16)	0.53 (0.31–0.90)^b^
Maternal health services		
Consumed 100 iron–folic acid		
No^a^		
Yes	0.56 (0.32–1.00)^b^	1.06 (0.83–1.34)
Taken drugs for intestinal worms		
No^a^		
Yes	1.58 (0.89–2.79)	0.85 (0.64–1.14)
Received supplementary food from Anganwadi Centre		
No^a^		
Yes	1.07 (0.64–1.81)	1.13 (0.89–1.43)
Received health and nutrition education at Anganwadi Centre		
No^a^		
Yes	1.79 (1.08–2.98)^b^	1.20 (0.95–1.51)
Dietary intake		
Consume daily milk/curd and pulses/beans or eggs/fish/meat and dark green leafy vegetables		
No^a^		
Yes	0.56 (0.27–1.17)	0.97 (0.71–1.31)
Eats fried food weekly		
No^a^		
Yes	0.87 (0.54–1.40)	0.95 (0.76–1.18)
Takes aerated drinks weekly		
No^a^		
Yes	1.57 (0.82–2.99)	1.03 (0.77–1.38)

Abbreviations: BAZ, body mass index for age *Z* score; NA, not applicable.

^a^ Reference category.

^b^ *P* < 0.05.

^c^ *P* < 0.01.

## 4 DISCUSSION

Our analysis of NFHS‐4 data shows that severe thinness affects <2% of the pregnancy cohort in India, which translates into 600 000 severely thin pregnant women out of 30 million pregnancies in the country annually. The burden is concentrated in a few districts. Three or more cases of severe thinness were found in 14 districts among pregnant women, and among mothers in 32 districts. The prevalence estimates from NFHS‐4 data matched findings from smaller research studies in community settings, but were much lower than estimates from facility‐based studies—the latter with more severe cases and those with medical comorbidities.

It is 25 years since WHO recommended the use of prepregnancy BMI and MUAC to screen pregnant women for thinness and severe thinness. Developing country‐specific cut‐offs for MUAC was also recommended.^19^ Evidence on MUAC cut‐offs is also available for India.^19^, ^21^, ^22^, ^23^ However, diagnosing thinness and severe thinness in pregnancy using these measures is still not integrated in India's public health system. Furthermore, these standard measures are not used consistently across research studies, making comparisons across studies challenging.

The nearly twice higher odds of severe thinness in adolescent pregnancies with an earlier parity compared with nulliparous women indicates a need to accelerate efforts to delay marriage and pregnancy in teenaged girls. Parity was not influential in increasing the odds of severe thinness in older mothers. Among diet‐related variables used in our model, the overall intake of a combination of foods (i.e. milk/milk products, protein‐rich foods, and dark green leafy vegetables) daily was low. It can explain the lack of association with severe thinness in pregnant adolescents and women. However, the lower odds of severe thinness among pregnant adolescents consuming fried foods weekly is a concern since it is indicative of possible “unhealthy” weight gain in this age group. In another UNICEF‐National Centre of Excellence and Advanced Research on Diets (NCEARD) report based on the Comprehensive National Nutritional Survey, it emerged that daily and weekly consumption of fried foods was high in the 15–19 years age group.^24^ Our findings also reveal that 36% of pregnant adolescents consumed fried foods weekly. Among mothers aged ≥20 years, level of education, poverty, and access to toilets influenced severe thinness. However, among severely thin mothers, usual *Anganwadi* services received during pregnancy revealed a correlation with increased odds of severe thinness among those receiving supplementary food and nutrition education. More needs to be done in terms of implementation research and service delivery of the usual package of services when pregnant women are severely thin.

## 5 CONCLUSION

The national prevalence of severe thinness was 4.3% in adolescents (15–19 years) and 1.9% in adult pregnant women (≥20 years), and 6.3% among postpartum adolescent women and 2.4% among postpartum adult women. Severe thinness was associated with earlier parity, level of education, poverty, access to toilets, household wealth, and caste. This article reveals the geographic pockets that need priority focus for managing severe thinness among pregnant adolescents and women to limit the immediate and intergenerational adverse consequences emanating from these deprivations.

## CONFLICTS OF INTEREST

The authors have no conflicts of interest.

## AUTHOR CONTRIBUTIONS

VS, AW and AB conceptualized the paper; TC drafted it with contributions from VS, AW. KD conducted the statistical analysis. All other authors reviewed the manuscript and contributed to the interpretation of findings. All authors agreed to the final version.
